# Prognostic utility of ^99m^Tc-MIBI single photon emission computerized tomography myocardial perfusion imaging in patients with ischemia and non-obstructive coronary artery disease

**DOI:** 10.3389/fcvm.2023.1115135

**Published:** 2023-07-04

**Authors:** Xiao-Hui Wang, Meng-Dan Li, Fu-Xiang Xie, Huan Liang, Lu Yang, Xiao-Fei Wei, Hua Pang, Zheng-Jie Wang, Xing-Guo Jing

**Affiliations:** Department of Nuclear Medicine, The First Affiliated Hospital of Chongqing Medical University, Chongqing Medical University, Chongqing, China

**Keywords:** SPECT, myocardial perfusion imaging, INOCA, MACE, prognosis

## Abstract

**Objective:**

The aim of our study was to evaluate the prognostic value of gated SPECT MPI in non-obstructed coronary arteries (INOCA) patients, sought to stratify patients more accurately and thus derive more reliable prognostic information.

**Materials and methods:**

In total, 167 patients with INOCA were enrolled. The patients were divided into two groups according to their SSS. Patients were followed-up regularly in terms of major adverse cardiovascular event (MACE), including cardiac death, nonfatal myocardial infarction, stroke, re-hospitalization with angina pectoris, and recurrent angina pectoris. Kaplan-Meier curves and Cox's proportional hazards models were used to analyze survival and identify predictive factors.

**Results:**

Adverse cardiac events occurred in 33 cases (19.8%). The rate of MACE was higher in the summed stress score (SSS) ≥4 group than in the SSS 0–3 group (30.1% vs. 9.5%, respectively, *P* = 0.001) and MACE-free survival was lower (annual MACE-free rates of 87.5% vs. 96.2%, respectively, *P* = 0.003). Event-free survival was consistently higher in patients with normal arteries than in those with non-obstructive coronary artery disease (annual MACE-free rates of 96.1% and 88.4%, *P* = 0.035). When the SSS and the CAG results were combined, patients with normal coronary arteries (SSS 0–3) had the best prognosis and those with non-obstructive coronary artery stenosis (SSS ≥ 4) had the worst. However, the early prognosis of patients with non-obstructive coronary artery disease and SSS of 0–3 was comparable to that of patients with normal coronary arteries and SSS ≥ 4 (annual MACE-free rates of 100%, 94.6%, 93.1%, and 78.2%, respectively). Multivariate Cox's regression indicated that the SSS [hazard ratio (HR) = 1.126, 95% confidence interval (CI) 1.042–1.217, *P* = 0.003] and non-obstructive coronary artery disease (HR = 2.559, 95% CI 1.249–5.246, *P *= 0.01) were predictors of adverse cardiac events.

**Conclusion:**

SPECT MPI data were prognostic for INOCA patients, thus identifying groups at high risk. The long-term predictive efficacy of such data exceeded that of CAG data. A combination of the two measures more accurately stratified INOCA patients in terms of risk.

## 1. Introduction

About 112 million people worldwide suffer from angina pectoris (1), which is the most common symptom of ischemic heart disease. In up to 70% of patients who undergo invasive coronary angiography (CAG) to treat angina pectoris, there is no evidence of obstructive coronary artery stenosis (stenosis < 50%) (2); their symptoms are often mistakenly considered non-cardiac in nature, resulting in inadequate follow-up treatment (3). CAG as the gold standard, aimed at the detection of obstructive CAD, whereas the other reasons for angina pectoris complaints are not investigated. Clinically, a disease with symptoms or signs of myocardial ischemia but without obstructive coronary artery stenosis evident in CAG is defined as ischemia of non-obstructive coronary arteries (INOCA). The clinical manifestations of INOCA are extensive and varied, and the symptom burden may change over time (4). Few studies have evaluated INOCA therapy and there is no evidence-based normative therapy (5). The symptoms are not specific and CAG does not reveal obstructive disease. If the doctor does not consider non-obstructive myocardial ischemia, the absence of obstructive coronary artery stenosis in CAG may trigger a misdiagnosis. Because they have not been appropriately diagnosed, such patients often complain of persistent chest pain and are likely to undergo repeated coronary angiography and other medical assessments, triggering physical limitations, a decline in their quality of life, anxiety, and depression (6). Ischemia changes can develop even without obvious severe coronary artery obstruction (7–9). Single photon emission computed tomography (SPECT) myocardial perfusion imaging (MPI) is the most reliable noninvasive method by which to detect myocardial ischemia (10–13); the technique directly examines coronary artery perfusion and determines the specific location, scope, and extent of ischemia (14). MPI accurately evaluates the prognosis of patients with coronary heart disease. Given the poor prognosis of some patients with INOCA, and the fact that symptoms of angina pectoris are associated with adverse events (15), might MPI usefully evaluate the prognosis of patients with INOCA, and accurately classify such patients, allowing selective interventions? Here, we divided angina pectoris patients with normal CAG data into different groups based on the results of SPECT MPI and CAG and explored the prognostic utility of SPECT MPI in patients with INOCA.

## 2. Materials and methods

### 2.1. Study population

This study complied with the Declaration of Helsinki and was approved by the ethical review board of the First Affiliated Hospital of Chongqing Medical University, Chongqing, China. Informed written consent was obtained from all patients. We enrolled all consecutive patients with non-obstructive coronary artery disease aged >18 years and hospitalized between June 1, 2017 and December 31, 2021, who met the following inclusion criteria: typical symptoms and signs of myocardial ischemia; <50% stenosis of the main branches of the coronary artery, as confirmed by CAG, including 0% stenosis; finish ^99m^Tc-MIBI SPECT stress and at rest myocardial perfusions after admission; and complete clinical data. The exclusion criteria were a history of coronary heart disease; any acute myocardial infarction (all ECGs were classified as normal or not myocardial infarction), acute cardiac insufficiency, valvular disease, congenital heart disease, or cardiomyopathy; musculoskeletal disease, chronic obstructive pulmonary disease, digestive system disease, mental disorders; a malignant tumor or severe liver or kidney disease; and expected survival for less than 1 year.

### 2.2. CAG results analysis

At the time of CAG, a cardiologist graded the severity of a coronary artery obstruction visually based on the percentage of stenosis, obtain an estimated value, then measurements are made on the screen to verify the accuracy of visual measurement, and the measured value shall prevail. Patients with major epicardial or branch vessels (diameter ≥2.0 mm) with <50% stenosis (including 0% stenosis) were classified as having either normal coronary arteries (0%) or non-obstructive coronary arteries (1%–49%) (16).

### 2.3. SPECT image acquisition and analysis

Patients were told to pause use of beta-blockers, calcium antagonists, nitrates, and any products containing caffeine for 24–48 h before SPECT. MPI featured respiratory gated^99m^Tc-sestamibi (^99m^Tc-MIBI) stress-rest alternate-day imaging. Stress testing was performed using the Bruce program and a treadmill exercise; the treadmill speed and slope were gradually increased until the heart rate attained 85% of the expected maximum (190 minus age) or when the patient exhibited angina pectoris, dyspnea, arrhythmia, a drop in blood pressure, or an ECG ST segment downshift >1 mm. Then, 740 MBq ^99m^Tc-MIBI was immediately injected intravenously and exercise continued for 1 min; 30 min later, patients were given a fatty meal. Stress myocardial tomography was performed at 90 min after injection. ECG data and blood pressure were monitored before, during, and after treatment. We used a GE Discovery SPECT/CT670 instrument equipped with a low-energy universal parallel hole collimator. The acquisition matrix was 128 × 128 from the right anterior oblique 45° point to the left posterior oblique 45° point. In total, 30 frames were collected over 40 s and the filtered back-projection method was used to reconstruct each image. A fatty meal was eaten 30 min after injection on the next day, and gated resting MPI was performed 90 min later. The stress and rest perfusion images were evaluated by at least two senior nuclear-medicine physicians using a double-blinded method based on a 17-segment left-ventricular model and a five-point scale (17) (0 = normal perfusion; 1 = mildly decreased, unable to determine whether abnormal; 2 = moderately reduced, abnormality evident; 3 = significant reduction, 4 = perfusion defect). If the two doctors did not agree, all doctors in the department were consulted and a consensus emerged. Perfusion scores were calculated to indicate myocardial perfusion abnormalities and their severity; the summed stress score (SSS) is the sum of all perfusion defects evident on the stress image, the summed rest score (SRS) is the sum of all defects apparent on the resting image, and the summed difference score (SDS) is the difference between the stress and rest scores. Left-ventricular (LV) functional parameters including the LV end-diastolic volume (EDV), LV end-systolic volume (ESV), and LV ejection fraction (LVEF) were calculated automatically by the QGS software. The patients were divided into an SSS 0–3 group and an SSS ≥ 4 group.

### 2.4. Follow-up

All patients were followed up every 6 months by our physicians, who contacted the patients or their family members to assess clinical status. We recorded major adverse cardiovascular event (MACE) including cardiac death, nonfatal myocardial infarction, stroke, re-hospitalization for angina pectoris, and recurrent angina pectoris. The survival time was from the time of MPI examination to the occurrence of the first MACE. If no MACE occurred during follow-up, the survival time was from the time of MPI examination to the end of follow-up.

### 2.5. Statistical analysis

All statistical analysis was performed using SPSS ver. 26.0 for Windows (SPSS Inc., Chicago, IL, USA). Continuous values are expressed as means ± SD or (median, inter quartile ranges) and categorical variables are given as percentages. The independent-samples *t*-test or Mann–Whitney *U*-test was used to compare numerical variables and the chi-square or Fisher exact test to compare categorical variables. The MACE-free survival rate was estimated by drawing Kaplan-Meier curves, and differences between survival curves were evaluated using the log-rank test. Univariate and multivariate Cox's proportional hazards models were employed to identify predictors of adverse events. Factors significant at *P* < 0.1 in univariate analysis were included in the multivariate analysis. We calculated hazard ratios (HRs) with 95% confidence intervals (CIs). Two-sided *P*-values < 0.05 were considered to indicate significance.

## 3. Results

### 3.1. Baseline clinical characteristics

In total, 167 patients were included. Table 1 shows the baseline data. There were 84 cases in the SSS 0–3 group (male/female 35/48) and 83 in the SSS ≥ 4 group (male/female 44/39). CAG showed that 102 patients had normal coronary arteries and 65 had non-obstructive coronary arteries. There were no significant differences in sex, age, or risk factors for coronary heart disease between the two groups. The proportion of patients with non-obstructive coronary arteries was higher in the SSS 0–3 group than in the SSS ≥ 4 group (*P* = 0.02), and such patients were less likely to be on an angiotensin-converting enzyme inhibitor/angiotensin II receptor (ACEI/ARB). There were no between-group differences in the use of aspirin, clopidogrel, beta-blockers, or calcium-channel blockers (CCBs).

**Table 1:** Comparison of baseline data between the two groups.

Variables	SSS 0–3 group (*n* = 84)	SSS ≥ 4 group (*n* = 83)	*P-*value
Demographics			
Age (years)	61.2 ± 8.68	59.07 ± 9.82	0.139
Female, *n* (%)	48 (57.1)	39 (47.0)	0.189
Risk factors			
Hypertension, *n* (%)	44 (52.4)	40 (48.2)	0.588
Diabetes, *n* (%)	18 (21.4)	24 (28.9)	0.265
Hyperlipidemia, *n* (%)	29 (34.5)	28 (33.7)	0.914
Smoking history, *n* (%)	26 (32.7)	28 (33.7)	0.824
Family history, *n* (%)	7 (8.6)	4 (4.8)	0.325
Non-obstructive coronary artery, *n* (%)	40 (47.6)	25 (30.1)	0.02
Medication			
Aspirin, *n* (%)	36 (42.9)	40 (48.2)	0.489
Clopidogrel, *n* (%)	23 (27.4)	23 (27.7)	0.962
ACEI/ARB, *n* (%)	21 (25.3)	37 (44.6)	0.009
Beta-blocker, *n* (%)	37 (44.0)	45 (54.2)	0.189
CCB, *n* (%)	19 (22.9)	22 (26.5)	0.589
Statin, *n* (%)	73 (86.9)	77 (92.8)	0.21

ACEI/ARB, angiotensin-converting enzyme inhibitor or angiotensin II receptor blocker; CCB, calcium channel blockers.

### 3.2. SPECT MPI results

Table 2 summarizes the SPECT MPI results. Compared to the SSS 0–3 group, LV function was significantly impaired in the SSS ≥ 4 group, showed with a lower LVEF and a higher end-diastolic volume (EDV) under both stress and rest. Patients with high SSS evidenced more severe myocardial perfusion damage and their LV function was affected accordingly.

**Table 2:** Comparison of SPECT data between the two groups (median, IQR).

Variables	SSS 0–3 group (*n* = 84)	SSS ≥ 4 group (*n* = 83)	*P-*value
Quantitative analysis index			
SSS	(0, 0)	(8, 7)	<0.001
SDS	(0, 0)	(3, 8)	<0.001
SRS	(0, 0)	(5, 4)	<0.001
Left ventricular functional parameters			
Stress LVEF (%)	(63, 11)	(59, 13)	0.027
Stress EDV (ml)	(75.5, 39)	(83, 45)	0.048
Stress ESV (ml)	(32, 32)	(33, 34)	0.306
Rest LVEF (%)	(64, 12)	(60, 15)	0.008
Rest EDV (ml)	(72, 41)	(80, 40)	0.054
Rest ESV (ml)	(30, 30)	(33, 30)	0.157

SSS, summed stress score; SRS, summed rest score; SDS, summed difference score; EDV, end-diastolic volume; ESV, end-systolic volume; LVEF, left ventricular ejection fraction.

### 3.3. Clinical outcomes

The average follow-up time was 24.15 ± 1.02 months. For the entire cohort, 33 MACEs (19.8%) occurred, including 2 cases (1.2%) of stroke, 12 cases (7.2%) of re-hospitalization to treat angina pectoris or heart failure, and 19 cases (11.4%) of angina pectoris recurrence, but no cardiac death or nonfatal myocardial infarction occurred. In the SSS 0–3 group of patients, eight MACEs (9.5%) occurred, including 1 case (1.2%) of stroke, 4 cases (4.8%) of re-hospitalization because of angina pectoris, and 3 cases (3.6%) of angina pectoris recurrence. In the SSS ≥ 4 group of patients, 23 MACEs (30.1%) occurred, including 1 case (1.2%) of stroke, 8 cases (9.6%) of re-hospitalization with angina pectoris, and 16 cases (19.3%) of angina pectoris recurrence. The incidence of MACE differed significantly between the two groups (*P* < 0.01) (Table 3).

**Table 3:** Comparison of incidence of MACE between the two groups (*n*, %).

Outcomes	SSS 0–3 group (*n* = 84)	SSS ≥ 4 group (*n* = 83)	*P-*value
Stroke	1 (1.2)	1 (1.2)	1
Re-hospitalization for angina pectoris or heart failure	4 (4.8)	8 (9.6)	0.222
Recurrent angina pectoris	3 (3.6)	16 (19.3)	0.001
Total	8 (9.5)	25 (30.1)	0.001

MACE, major adverse cardiac event; SSS, summed stress score.

**Figure 1:**
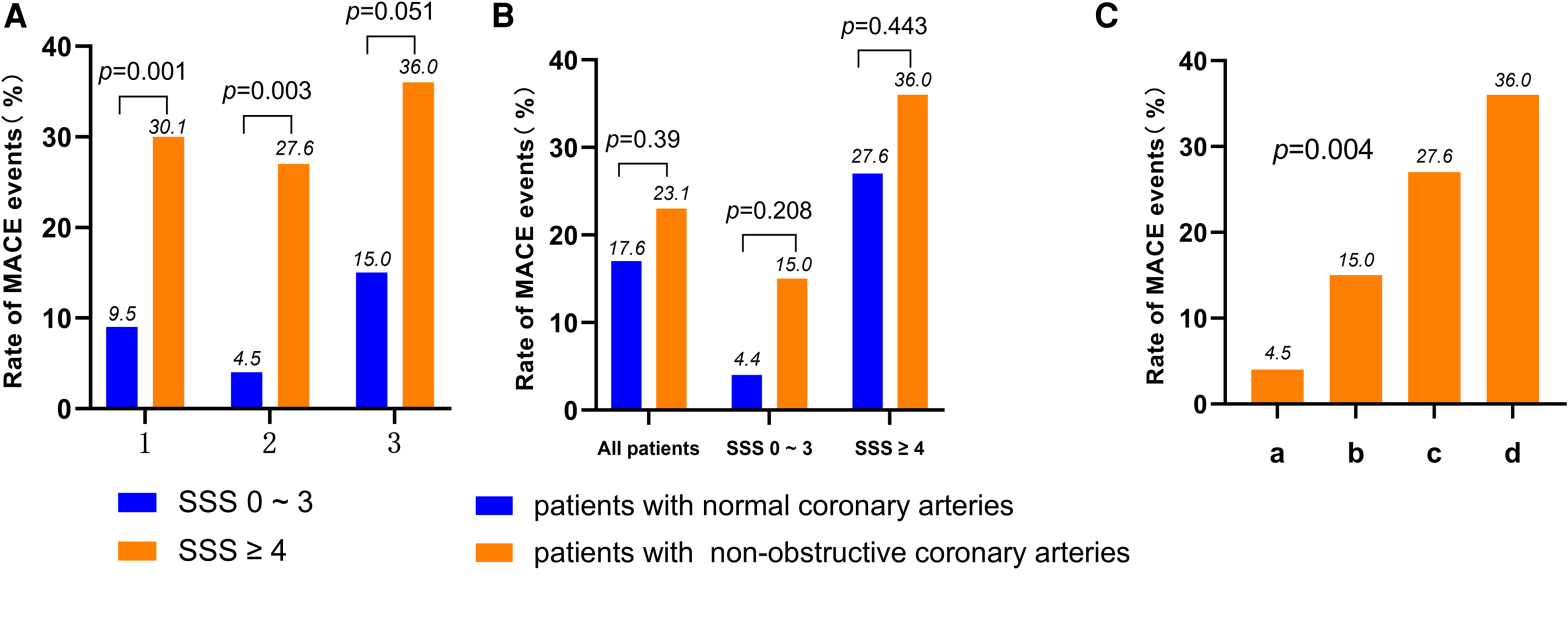
(**A**) Rate of MACE according to the result of SSS in all patients, normal coronary arteries patients and non-obstructive coronary arteries patients (1: *all patients*; 2: *normal coronary arteries patients*; 3: *non-obstructive coronary arteries patients*). (**B**) Rate of MACE according to the result of CAG in all patients, SSS 0–3 score patients and SSS ≥ 4 score patients. (**C**) Rate of MACE according to the result of SSS and the result of CAG in all patients (a: *patients of normal coronary arteries with SSS 0–3*; b: *patients of non-obstructive coronary arteries with SSS 0–3*; c: *patients of normal coronary arteries with SSS ≥ 4*; d: *patients of non-obstructive coronary arteries with SSS ≥ 4*). MACE, major adverse cardiac events; MPI, myocardial perfusion imaging; CAG, coronary angiography; SSS, summed stress score.

As shown in Figure 1A, in the patients with normal coronary arteries, the incidences of MACE in the SSS 0–3 and SSS ≥ 4 groups were 4.5% and 27.6%, respectively (*P *= 0.003); in those with non-obstructive coronary arteries, the incidences in the SSS 0–3 and SSS ≥ 4 groups were 15.0% and 36.0%, respectively (*P *= 0.051). CAG revealed that the incidences of MACE in patients with normal and non-obstructive coronary arteries were 17.6% and 23.1%, respectively (*P *= 0.39). In the SSS 0–3 group, the incidences of MACE were 4.5% in patients with normal coronary arteries and 15% in those with non-obstructive coronary arteries (*P *= 0.208), while in the SSS ≥ 4 group, the incidences were 27.6% and 36%, respectively (*P *= 0.443) (Figure 1B). Thus, the higher the SSS, the higher the MACE incidence. CAG revealed that although the incidence of MACE was higher in the patients with non-obstructive coronary arteries than in those with normal arteries, this difference did not attain statistical significance. SSS were more closely related to the incidence of MACE than was non-obstructive coronary artery status. Figure 1C shows the occurrence of MACE predicted by combining SSS and CAG results (*P *= 0.004).

### 3.4. Survival analysis

**Figure 2:**
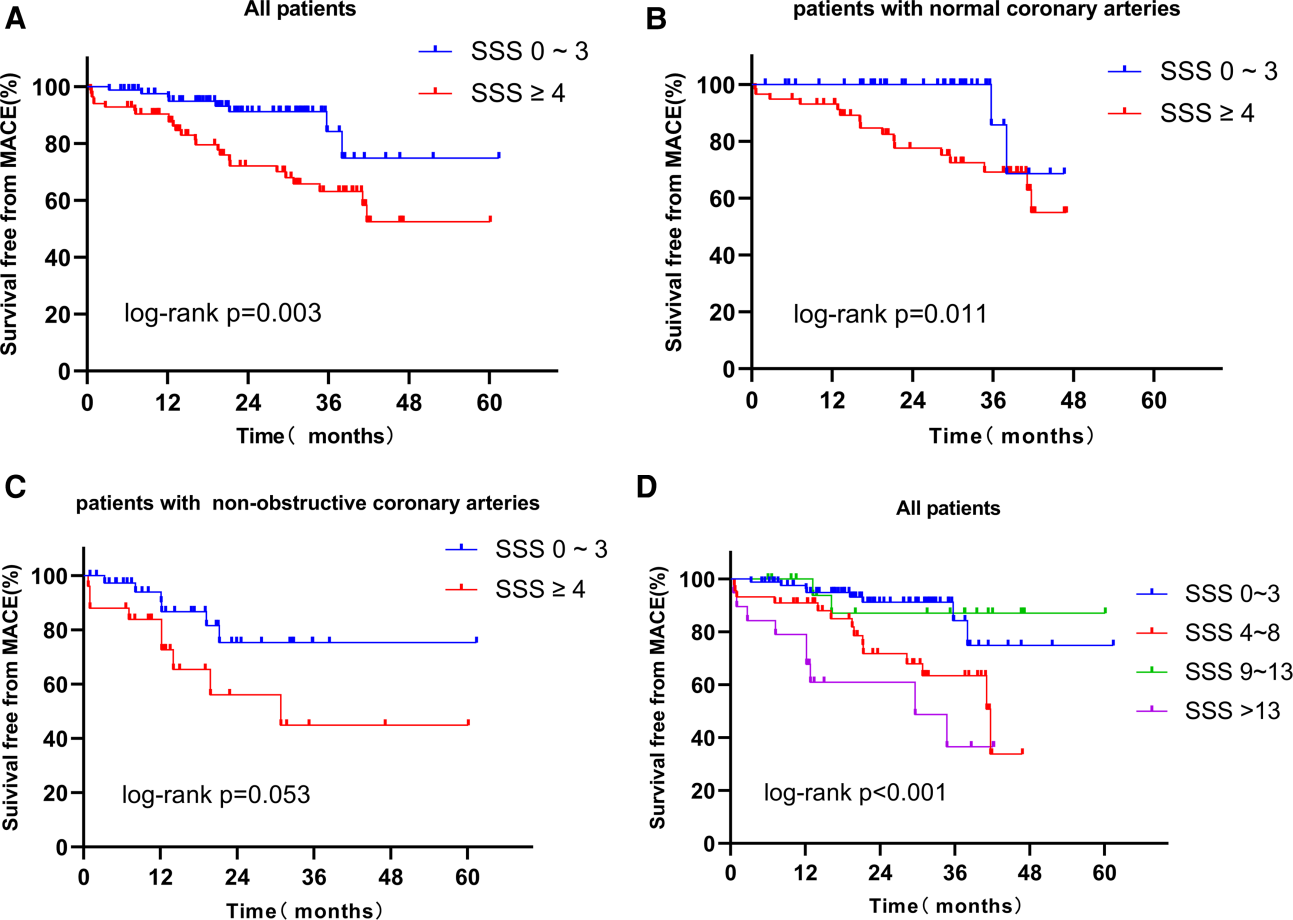
(**A**) Kaplan-Meier curves for freedom from MACE according to the result of SSS in all patients. (**B**) Kaplan-Meier curves for freedom from MACE according to the result of SSS in patients with normal coronary arteries. (**C**) Kaplan-Meier curves for freedom from MACE according to the result of SSS in patients with non-obstructive coronary arteries. (**D**) Kaplan-Meier curves for freedom from MACE according to the result of extent of ischemia myocardial (*Group 1: SSS 0–3: no myocardial ischemia; Group 2: SSS 4–8, mild myocardial ischemia; Group 3: SSS 9–13, moderate myocardial ischemia; Group 4: SSS > 13, severe myocardial ischemia*). MACE, major adverse cardiac event; SSS, summed stress score.

Figure 2A shows the Kaplan-Meier survival curves of the SSS 0–3 and SSS ≥ 4 groups; the respective survival rates for 1, 2, 3, and 4 years were 96.2% and 87.5%, 91.2% and 72.1%, 84.1% and 63.1%, and 74.8% and 52.5%. In survival analysis of the normal coronary artery patients and the non-obstructive coronary artery patients, the survival rates of the SSS ≥ 4 patients were always lower (Figures 2B,C). Survival analysis was performed for all patients, stratified by the severity of myocardial ischemia (group 1: SSS 0–3; group 2: SSS 4–8; group 3: SSS 9–13; and group 4: SSS > 13). The annual MACE-free rates of groups 1, 2, 3, and 4 were 96.2%, 90.9%, 100%, and 78.6%, respectively (Figure 2D). A strong correlation was evident between SSS and the prognosis of INOCA patients: the higher the SSS, the lower the survival rate.

**Figure 3:**
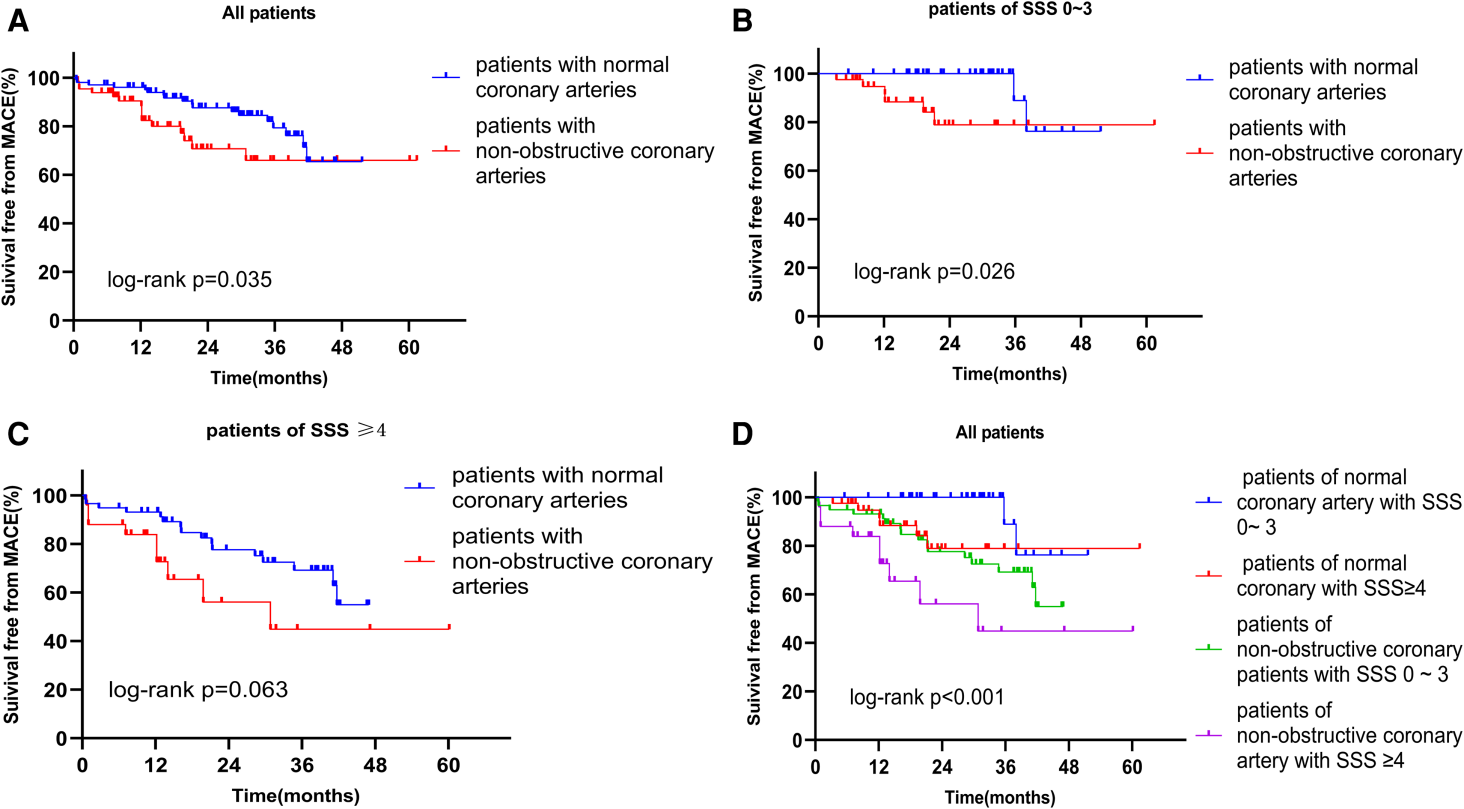
(**A**) Kaplan-Meier curves for freedom from MACE according to the result of CAG in all patients. (**B**) Kaplan-Meier curves for freedom from MACE according to the result of CAG in patients of SSS 0–3. (**C**) Kaplan-Meier curves for freedom from MACE according to the result of CAG in patients of SSS ≥ 4. (**D**) Kaplan-Meier curves for freedom from MACE according to the result of SSS and the result of CAG. MACE, major adverse cardiac event; SSS, summed stress score; CAG, coronary angiography.

Based on the CAG data, we performed survival analysis of all patients, SSS 0–3, and SSS ≥ 4 patients. The respective survival rates of the normal and non-obstructive coronary artery groups for 1, 2, and 3 years were 96.1% and 88.4%, 87.6% and 70.7%, and 79.3% and 66.0% (Figure 3A). Regardless of grouping, survival rates were always higher in patients with normal coronary arteries, indicating that obstruction significantly affected the survival of patients with INOCA (Figures 3B,C). Based on Kaplan-Meier curves obtained by combining SSS and angiographic results, the annual MACE-free rates of the four groups were 100%, 94.6%, 93.1%, and 78.2%, respectively (Figure 3D). The prognosis of the normal coronary artery patients with SSS of 0–3 was the best, and that of non-obstructive coronary artery patients with SSS ≥ 4 was the worst. Thus, a combination of the SPECT MPI and CAG data predicted the prognosis of patients with INOCA more accurately.

### 3.5. Analysis of prognostic factors

To identify factors predictive of MACE, we performed Cox's univariate and multivariate regression analyses. In univariate analysis, a non-obstructive coronary artery (*P *= 0.004), the SSS (*P *= 0.039), SRS (*P *= 0.002), and stress EDV (*P *= 0.088) were risk factors for MACE. Factors with *P-*values < 0.1 were included in the multivariate model. The SSS (HR = 1.126, 95% CI 1.042–1.217) and a non-obstructive coronary artery (HR = 2.559, 95% CI 1.249–5.246) were risk factors for MACE, but the predictive effect of the SSS was higher than that of non-obstructive coronary artery status (Table 4).

**Table 4:** Univariate and multivariate COX regression analysis of MACE.

	Univariate		Multivariate	
	HR (95% CI)	*P-*value	HR (95% CI)	*P-*value
Age	1.019 (0.984–1.056)	0.291		
Sex	1.078 (0.554–2.136)	0.829		
Hypertension	0.777 (0.389–1.552)	0.475		
Diabetes	1.08 (0.501–2.327)	0.844		
Hyperlipidemia	0.555 (0.240–1.280)	0.167		
Smoking history	1.016 (0.483–2.138)	0.966		
Family history	0.879 (0.21–3.684)	0.86		
Non-obstructive coronary artery	2.082 (1.037–4.183)	0.039	2.559 (1.249–5.246)	0.01
Aspirin	0.834 (0.417–1.669)	0.608		
Clopidogrel	1.584 (0.749–3.351)	0.229		
ACEI/ARB	0.858 (0.408–1.804)	0.687		
Beta-blocker	0.902 (0.454–1.791)	0.769		
CCB	0.927 (0.402–2.136)	0.858		
Statin	1.465 (0.446–4.813)	0.529		
SSS	1.075 (1.03–1.122)	0.001	1.126 (1.042–1.217)	0.003
SRS	1.007 (1.012–1.145)	0.018	0.912 (0.791–1.052)	0.207
SDS	1.126 (1.044–1.214)	0.102		
Stress LVEF	0.981 (0.956–1.007)	0.152		
Stress EDV	1.005 (0.999–1.012)	0.088	1.004 (0.996–1.012)	0.288
Stress ESV	1.006 (0.999–1.013)	0.12		
Rest LVEF	1.007 (0.977–1.039)	0.64		
Rest EDV	1.004 (0.998–1.011)	0.185		
Rest ESV	1.004 (0.996–1.012)	0.318		

ACEI/ARB, angiotensin-converting enzyme inhibitor or angiotensin II receptor blocker; CCB, calcium channel blockers; SSS, summed stress score; SRS, summed rest score; SDS, summed difference score; EDV, end-diastolic volume; ESV, end-systolic volume; LVEF, left ventricular ejection fraction; HR, hazard ratio; CI, confidence interval.

## 4. Discussion

Our study investigated the prognostic value of MPI in patients with INOCA, the data show that MPI was highly and independently predictive of MACE, moreover, INOCA patients with non-obstructive coronary artery have a worse prognosis than those with normal coronary artery, which contributes to our unfolding knowledge regarding the adverse event risk associated with INOCA patients.

Although there have been many studies on INOCA, the pathogenesis, diagnosis, treatment, and prognosis of stable chest pain and INOCA remain very controversial (15). With the CAG either obstructions are detected or in some cases spasms. In case the obstructions are not severe enough to limit the blood flow severely, the CAG will be classified as minor obstructive or maybe also normal. SPECT MPI May be a good tool to distinguish local changes in the heart. We used MPI to evaluate INOCA prognoses; after a follow-up of 24.15 ± 1.02 months, about half (83/167) of all patients evidenced abnormal MPI data (SSS ≥ 4). The incidence of MACE was 3.2-fold higher in the SSS ≥ 4 group than in the SSS 0–3 group. For patients with normal coronary arteries, the incidence was 6.1-fold higher in the SSS ≥ 4 group than in the SSS 0–3 group; for those with non-obstructive coronary arteries, the incidence was 2.4-fold higher in the SSS ≥ 4 group than in the SSS 0–3 group. When the SSS and CAG data were combined, patients with an SSS ≥ 4 and non-obstructive coronary artery status evidenced the highest incidence of MACE (36%).

**Figure 4:**
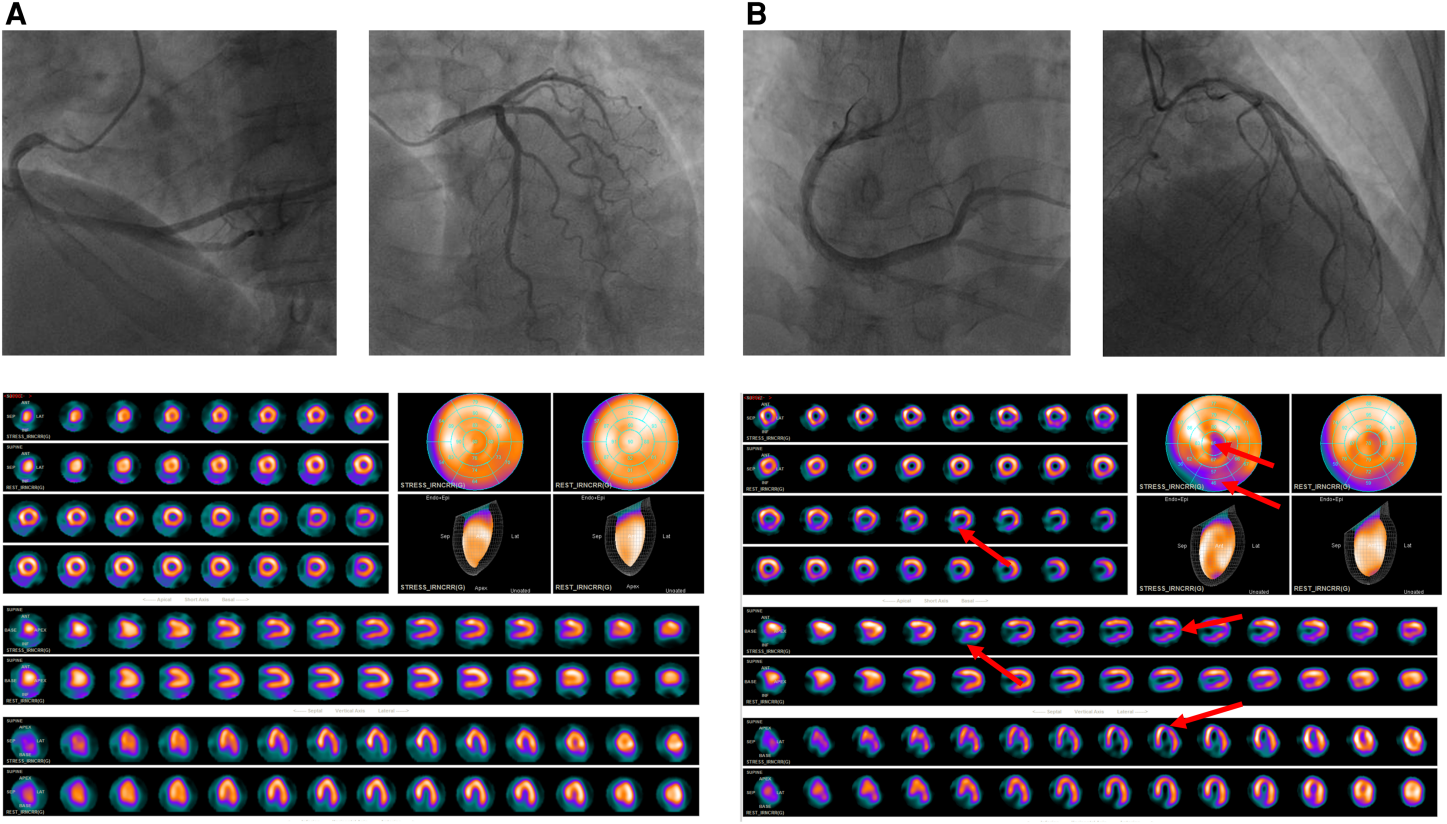
(**A**) A 60-year-old female patient presented to the cardiology department of our hospital for “chest pain for 2 + months”, CAG suggested that there was no stenosis in the left and right coronary arteries; SPECT MPI was normal, the SSS = 0, by the follow-up date of 25 months, the patient did not have MACE (**B**) A 52-year-old female patient presented to the cardiology department of our hospital for “repeated chest pain for 6 days and aggravation for 11 h”, CAG suggested that there was no stenosis in the left and right coronary arteries; SPECT MPI indicates sparse radioactive distribution of left ventricular part of the segments in stressing phase (red arrow), and filling in resting phase, considering myocardial infarction, the SSS = 9, the patient was re-hospitalized 8 months later due to angina pectoris.

All included patients with chest pain were normal in CAG but almost half evidenced abnormal SPECT MPI results (Figure 4) and were at increased risk for adverse cardiovascular outcomes. The reason may be that such patients suffer from coronary microvascular disease and/or epicardial coronary artery spasms that mismatch coronary blood supply and demand (8). Many recent studies have confirmed that coronary microvascular dysfunction is associated with an increased risk for adverse cardiac outcomes in patients with INOCA (18, 19). No existing technique directly observes the anatomy of the human coronary artery microcirculation. We found that the incidences of MACE in patients with SSS of 0–3 and SSS ≥ 4 were 4.5% and 27.6%, respectively, in the whole cohort, without myocardial infarction and death, comparable to those reported by Alqaisi (20) and Liu et al. (21), this indicates that MACE events in INOCA patients are better than obstructive CAD. The multivariate proportional hazard model showed that every unit increase in the SSS increased the MACE risk by 12.6%. Thus, even if there is no obstructive coronary artery disease, the probability of MACE in patients with abnormal SPECT MPI data is still significant, and their prognosis is often worse. All patients were further grouped by their SSS (a measure of ischemia severity). The Kaplan-Meier curve showed that the prognosis of the non-ischemia group was the best, and that of the severe ischemia group was the worst, similar to the results of previous studies (22, 23), indicating that the prognosis of INOCA patients is affected by myocardial ischemia and that survival becomes increasingly poorer as ischemia becomes aggravated. Unexpectedly, the prognosis of patients with moderate ischemia was better than that of those with mild ischemia, possibly for the reason: after patients with SSS ≥ 4 were re-grouped, the SSS 9–13 group had the lowest number of patients and the least MACE, so it showed the best long-term prognosis on the survival curve. SPECT MPI is demanding in terms of equipment, and on patients and operators; it is less popular than CAG in clinics. Many institutions perform only CAG. However, if patients have clinical symptoms of coronary heart disease, particularly angina pectoris, these should not be taken lightly even if CAG reveals no obstructive coronary heart disease. SPECT MPI evaluates myocardial ischemia status and its severity, assisting in treatment decisions. Interestingly, COX regression analysis showed that SDS was not an independent predictor of MACE, this may be a proportion of our included patients had an irreversible myocardial ischemia, which affected its predictive function.

Multivariate regression analysis also showed that the risk for MACE was 2.6-fold higher in patients with non-obstructive coronary arteries than in patients with normal coronary arteries; we thus further examined the prognosis and survival of such patients. Using the CAG data, all patients, and patients with SSS of 0–3 and ≥4, were divided into normal and non-obstructive coronary artery groups. The survival rates of the non-obstructive coronary artery groups were always lower than those of the other groups. Jespersen et al. (15) showed that, compared to a reference population with no ischemic heart disease, patients with normal coronary arteries but symptoms of angina pectoris, and patients with non-obstructive coronary arteries but symptoms of angina pectoris have an increased risk of MACE by 52% and 85% respectively, and as the extent of vascular stenosis rose, the risk for MACE and mortality from various causes gradually increased. Another study (24) showed that the risks for repeat CAG examination in patients with normal and non-obstructive coronary arteries were 2.3% and 5.5%, respectively. For each additional segment of non-obstructive coronary artery disease, the mortality rate increased by 6% (95% CI 1%–12%, *P *= 0.021). Both our findings and those described above indicate that even when coronary artery stenosis is less than 50% in INOCA patients, non-obstructive coronary lesions predict an increased risk for adverse cardiovascular outcomes, perhaps because the endothelial function of non-obstructive coronary arteries is impaired and development of atherosclerotic coronary artery disease is thus more likely (25), and/or a non-obstructive coronary artery features diffuse non-obstructive atherosclerosis with “compensatory” coronary remodeling (26) and the normal structure has thus been damaged. For such patients, the rates of re-hospitalization and repeat angiography caused by increasingly problematic symptoms rise, adding to the economic and psychological burdens on patients and their families, social medical pressure, and economic costs. Clinicians must strive to avoid such negative effects.

We combined SSS and CAG data to analyze the survival of all patients. The survival curves showed that the early prognosis of non-obstructive coronary artery patients with SSS of 0–3 was similar to that of normal coronary artery patients with SSS ≥ 4, but over time the prognosis of the latter patients was poorer than that of the former. In other words, in the long run, the predictive power of SPECT MPI was higher than that of CAG. The best prognosis was that for patients with normal coronary arteries and SSS of 0–3 (annual survival 100%), whereas patients with SSS ≥ 4 had the worst prognosis (annual survival 78.2%); such patients require much more attention than they presently receive. In institutions that prefer CAG, non-obstructive coronary artery lesions should not be ignored; SPECT MPI is essential. A combination of the data of both examinations increased the prognostic accuracy for INOCA patients.

In addition, we found that the proportion of non-obstructive coronary artery patients was higher in the SSS 0–3 group than in the SSS ≥ 4 group. In other words, there was no direct correlation between SSS and the extent of coronary artery stenosis in the INOCA patients. Coronary atherosclerosis was not the principal cause of myocardial ischemia in the INOCA patients.

Some limitations to our study should be mentioned. First, we found that drugs did not improve the INOCA prognosis, whereas previous studies have found that statins significantly reduced the incidence of MACE in INOCA patients (16). This could have been because of drug non-compliance by our patients, which may have affected our findings. Second, our sample size was small; more patients are required to draw more accurate conclusions.

Secondly, the definite diagnosis of the origin of INOCA is based on coronary function tests with for instance acetylcholine or adenosine to elucidate the true origin, but the patients in this study did not have coronary functional testing, in a future study, we do this analysis with patients undergoing such test. In addition, this was a retrospective single-center study and the results are thus not necessarily generally applicable. There may have been some selection bias. Larger multicenter studies are needed to verify our conclusions. Our average follow-up time was short. Some patients did not develop MACE; cardiovascular deaths and nonfatal myocardial infarctions were rare.

Importantly, we not only confirmed the prognostic utility of SPECT MPI in patients with INOCA but also combined SPECT MPI and CAG data to develop a new and accurate risk-stratification method for INOCA patients. We found that SPECT MPI yielded valuable prognostic information on INOCA patients, identifying those at higher risk. By measuring the response to myocardial perfusion, SPECT MPI identifies myocardial ischemia, and its location and severity, and also comprehensively evaluates coronary artery anatomy and function. The long-term predictive efficacy of the data is even higher than that of CAG data. The combination of the two datasets enhanced the accuracy of INOCA patients' risk stratification.

The English in this document has been checked by at least two professional editors, both native speakers of English. For a certificate, please see: http://www.textcheck.com/certificate/5dUdCx.

## Data Availability

The original contributions presented in the study are included in the article/Supplementary Material, further inquiries can be directed to the corresponding authors.
